# Supplementary material for: Distribution of ascariasis, trichuriasis and hookworm infections in Ogun State, Southwestern Nigeria
Source: PLoS One. 2020 Jun 8;15(6):e0233423. doi: 10.1371/journal.pone.0233423 (PMC7279584; doi:10.1371/journal.pone.0233423)
Supplement: Attachment — Submitted filename: Comments on the reviewed MS.docx [file pone.0233423.s002.docx]

**Comments on the reviewed MS**

General comments

The study was properly designed and well executed. Nigeria is an endemic country for soil-transmitted helminthiases and there are still some regions that lack epidemiological data on the prevalence of STH, hence, the results from this comprehensive study will provide additional data which will assist the public health officers to institute an appropriate control strategy.

Specific comments

**Abstract**

Result section

Line 2-4: The sentences “*Ascariasis was* the most spatially distributed found in 28 communities, with 140(13.6%) infected subjects, followed byHookworm infection in 19 communities, with 47(4.6%) infected subjects and *Trichuriasis* in 9 communities, with 17(1.7%) infected subjects.”

should be reconstructed to read “ Ascariasis was the most frequently observed parasite in 28 communities with a prevalence of 13.6%, followed by hookworm with a prevalence of 4.6% while Trichuris was the least encountered with a prevalence of 1.7%.

Line 6-7: The sentence is incomplete “The highest and lowest distribution of what (?) was recorded in Yewa South and Yewa North respectively.

Line 8: Delete the phrase “By WHO preventive chemotherapy thresholds”

Conclusion

This study provides information on the prevalence and spatial risk of Ascariasis, Trichuriasis

and Hookworm infection that will serve as decision-support tool to help facilitate targeting of

control interventions.

The conclusion should be re-written as “ This study provides epidemiological data on the prevalence and spatial distribution of ascariasis , trichuriasis and hookworm infection which will add to the baseline data and guide the public health officers in providing appropriate control strategies in the endemic communities.

**Background**

Line 7-8: Replace the phrases in parentheses (children between age 2 and 5) and (children between age 5 and 14) with (age 2-5 years) and (age 5-14 years).

Line 15: The sentence “there is an established morbidity control programme targeted at school-aged children due to their high level of contact with soils” should be re-written as “there is an established morbidity control programme targeted at school-aged children due to high worm burden as a result of their frequent contact with contaminated soils, ……………………….

**Methods**

Study area

Line 10: The phrase “The major occupation of the population is……………… should be re-phrased as “Major occupations of the population are………………

Study design and sampling technique

Line 1: The sentence “This study was community-based and cross-sectional in design” should be re-written as “This is a cross-sectional and community-based study”.

Selection of households for survey

Line 2: The sentence “communities were visited and sensitized about the study procedures

and were openly invited to participate in the research through community meetings and town

announcers” should be replaced with “selected communities were visited and with the permission of the community leaders, meetings were held with the members of the community to intimate them about the purpose of the study and the procedures to be adopted”.

The sub title “**Collection of faecal samples and parasitological diagnosis”** be replaced with **“ Faecal samples collections and processing”.**

Line 3-4: The sentence “All participants were asked to provide a sufficiently large stool sample (at least 5 g)” should be replaced with “Individuals who consented to take part in the study were requested to provide stool up to half (about 5gm) into the labelled plastic bottles provided.

Line 6: Delete the word “Precisely”.

Line 7-8: The sentence “The bottle was covered and agitated vigorously to suspend the stool efficiently in the solution” be replaced with “The bottle was covered and shaken vigorously to form a cloudy suspension”.

Line 9: Delete the word “further”

Line 11: The sentence “The supernatant was also discarded after centrifuging” be replaced with “The supernatant was decanted”.

Line 12-14: The sentence “7 ml of normal saline was later added to the sediment, after which 3 ml of ether was finally added to the suspension”. ( I did not agree with this procedure, it is faulty). The deposit or residue obtained after decanting the supernatant should be resuspended in distilled water or saline of a known vol and shaking vigorously. Thereafter, an equal vol of ether will then be added (i.e if 3ml of distilled water or saline was used to resuspend the residue, 3ml of ether should then be added). The authors should correct this procedure.

**Results**

Sex and age distribution of study participants

The sentence “A total of 1,499 respondents were subsequently enrolled after consent across the 33 communities and 20 LGAs. However, only 1,027 (68.5%) of them consented to the provision

of adequate stool samples (~5g) for laboratory analysis for parasites’ ova or larva. By

demography, 899(60.0%) were females and 600(40.0%) were males” should be rewritten as “ A total of 1499 participants comprising of 899 (60%) females and 600 (40%) males were enrolled across the 33 communities in 20LGAs out of which 1024 (68.5%) returned stool samples for laboratory analysis.

The sentence “The sex ratio of females to males was 3:2. In addition, the highest number of respondents were within the age range 41-70yrs (40.6%), followed by 26-40yrs (30.3%), 5-15yrs (10.7%), 16-25yrs (10.4%) and >70yrs (7.0%) (Table 2)” should be re-written as “The ages of the enrolled participants are provided in table 2.

**Co-distribution patterns of *Ascaris*, *Trichuris* and Hookworm infections in Ogun State**

Line1: Replace the words “mono infection” with single infection

Line 2: Delete the word “only”

Line 3: Replace the word “co-distribution” with the word “combination”

Line 5: Begin with combination of Ascaris and Trichuris and delete the word “co-distribution”

Line 6 & 7: Rewrite “while *Trichuris* and *Hookworm* co-distribution was recorded in 6 LGAs;” as “while combination of Trichuris and hookworm was……………………….”

Line 8-10: Rewrite “However, for triple infections, the three species (*Ascaris*, *Trichuris* and Hookworm) were co-distributed in 6 LGAs; Ado-Odo, Ipokia, Obafemi-Owode, Ogun waterside, Yewa North and Yewa South” as “ Triple infection comprising Ascaris, Trichuris and hookworm was recorded in 6 LGAs (table 3)”.

**Aggregated prevalence of *Ascaris*, *Trichuris* and Hookworm infections in Ogun State**

The sentences “The aggregated prevalence of 17.2% was recorded for at least one infection of *Ascaris lumbricoides*, *Trichuris trichiura* or hookworm. The prevalence range was between 5.3 –

49.2% across the LGAs (Figure 4). Infections were highest in Yewa south, lowest in Yewa

north and no infection was recorded in Ijebu-Ode. Of the 19 endemic LGAs, 9 had prevalence

status ranging between 20.0%-49.2% (Abeokuta north, Abeokuta south, Ifo, Ikenne, Ipokia,

Obafemi owode, Ogun waterside, Remo north and Yewa south” **should be reduced** to “ The highest prevalence of 49.2% was recorded in Yewa South while the lowest of 5.3% was recorded in Yewa North, however, no infection was recorded in Ijebu-Ode.

**Delete** this sentence “However, 10 LGAs haprevalence status ranging between 5.3%-15.8% which is below the WHO recommended thresholds for preventive chemotherapy (Ado-odo ota, Ewekoro, Ijebu east, Ijebu north, Ijebu north east, Imeko afon, Odeda, Odogbolu, Sagamu and Yewa north).

**Specific prevalence estimates for *Ascaris*, *Trichuris* and Hookworm infections in Ogun**

**State.**

The contents under this section is too wordy. The authors should highlight significant findings in terms of the most frequently encountered parasite in each LGA, the range of other parasites and if there is significant differences in prevalence of infection across the communities.

**Aggregated and specific mean intensity estimates for *Ascaris*, *Trichuris* and Hookworm**

**infections in Ogun State.**

The authors should express the intensity as eggs per gram (epg) of faeces i.e epg should be put in front of the mean intensity values in all cases.
