# Supplementary material for: Distribution of ascariasis, trichuriasis and hookworm infections in Ogun State, Southwestern Nigeria
Source: PLoS One. 2020 Jun 8;15(6):e0233423. doi: 10.1371/journal.pone.0233423 (PMC7279584; doi:10.1371/journal.pone.0233423)
Supplement: Attachment — Submitted filename: Response to Reviewers_2.docx [file pone.0233423.s003.docx]

PONE-D-20-06434
Spatial Distribution of Ascariasis, Trichuriasis and Hookworm Infections in Ogun State, Southwestern Nigeria.
PLOS ONE

Reviewer 2 Comments:

Reviewer's Responses to Questions

**Comments to the Author**

1. Is the manuscript technically sound, and do the data support the conclusions?

The manuscript must describe a technically sound piece of scientific research with data that supports the conclusions. Experiments must have been conducted rigorously, with appropriate controls, replication, and sample sizes. The conclusions must be drawn appropriately based on the data presented.

Reviewer #2: Yes

2. Has the statistical analysis been performed appropriately and rigorously?

Reviewer #2: Yes

3. Have the auhors made all data underlying the findings in their manuscript fully available?

The [PLOS Data policy](http://www.plosone.org/static/policies.action#sharing) requires authors to make all data underlying the findings described in their manuscript fully available without restriction, with rare exception (please refer to the Data Availability Statement in the manuscript PDF file). The data should be provided as part of the manuscript or its supporting information, or deposited to a public repository. For example, in addition to summary statistics, the data points behind means, medians and variance measures should be available. If there are restrictions on publicly sharing data—e.g. participant privacy or use of data from a third party—those must be specified.

Reviewer #2: No

4. Is the manuscript presented in an intelligible fashion and written in standard English?

PLOS ONE does not copyedit accepted manuscripts, so the language in submitted articles must be clear, correct, and unambiguous. Any typographical or grammatical errors should be corrected at revision, so please note any specific errors here.

Reviewer #2: Yes

5. Review Comments to the Author

Please use the space provided to explain your answers to the questions above. You may also include additional comments for the author, including concerns about dual publication, research ethics, or publication ethics. (Please upload your review as an attachment if it exceeds 20,000 characters)

Reviewer #2: Specific comments

Abstract

Result section

C1: Line 2-4: The sentences “Ascariasis was the most spatially distributed found in 28 communities, with 140(13.6%) infected subjects, followed by Hookworm infection in 19 communities, with 47(4.6%) infected subjects and Trichuriasis in 9 communities, with 17(1.7%) infected subjects.” should be reconstructed to read “ Ascariasis was the most frequently observed parasite in 28 communities with a prevalence of 13.6%, followed by hookworm with a prevalence of 4.6% while Trichuris was the least encountered with a prevalence of 1.7%.

R1: We have modified our text as suggested. Please find the improved text in between lines 45-48. Thanks

C2: Line 6-7: The sentence is incomplete “The highest and lowest distribution of what (?) was recorded in Yewa South and Yewa North respectively.

R2: We have improved our text.

Line 49: The highest and lowest distribution of overall helminth infections was recorded in Egbado South and Egbado North respectively

C3: Line 8: Delete the phrase “By WHO preventive chemotherapy thresholds”

R3: The phrase has been deleted as suggested.

Line 50-51: Nine regions had infection status between 20.0%-49.2%, while 10 regions had infection status between 5.3%-15.8%.

**Conclusion**

C4: This study provides information on the prevalence and spatial risk of Ascariasis, Trichuriasis and Hookworm infection that will serve as decision-support tool to help facilitate targeting of control interventions.

The conclusion should be re-written as “ This study provides epidemiological data on the prevalence and spatial distribution of ascariasis , trichuriasis and hookworm infection which will add to the baseline data and guide the public health officers in providing appropriate control strategies in the endemic communities.

R4: We have modified our text as suggested. Please find the improved text in between lines 53-56. Thanks

Line 53-56: This study provides epidemiological data on the prevalence and spatial distribution of ascariasis , trichuriasis and hookworm infections which will add to the baseline data and guide the public health officers in providing appropriate control strategies in the endemic communities.

**Background**
C5: Line 7-8: Replace the phrases in parentheses (children between age 2 and 5) and (children between age 5 and 14) with (age 2-5 years) and (age 5-14 years).

R5: We have modified our text as suggested. Please find the improved text in between lines 66-67. Thanks

Line 66-67: Besides, about 267 million pre-schoolers (age 2-5 years) and 568 million school-aged children (age 5-14 years) who live in areas where these

C6: Line 15: The sentence “there is an established morbidity control programme targeted at school-aged children due to their high level of contact with soils” should be re-written as “there is an established morbidity control programme targeted at school-aged children due to high worm burden as a result of their frequent contact with contaminated soils, ……………………….

R6: We have modified our text as suggested. Please find the improved text in between lines 72-74. Thanks

Lines 72-74: Nonetheless, in most endemic settings, there is an established morbidity control programme targeted at school-aged children due to high worm burden as a result of their frequent contact with contaminated soils


**Methods
Study area**

C7: Line 10: The phrase “The major occupation of the population is……………… should be re-phrased as “Major occupations of the population are………………

R7: We have modified our text as suggested. Please find the improved text in between lines 96. Thanks

Line 96: Major occupations of the population are farming, timber logging, and trading.

**Study design and sampling technique**
C8: Line 1: The sentence “This study was community-based and cross-sectional in design” should be re-written as “This is a cross-sectional and community-based study”.

R8: We have modified our text as suggested. Please find the improved text in between lines 101-102. Thanks

Line 101-102: This is a cross-sectional and community-based study, conducted between July 2016 and November 2018.

Selection of households for survey

C9: Line 2: The sentence “communities were visited and sensitized about the study procedures and were openly invited to participate in the research through community meetings and town announcers” should be replaced with “selected communities were visited and with the permission of the community leaders, meetings were held with the members of the community to intimate them about the purpose of the study and the procedures to be adopted”.

R9: We have modified our text as suggested. Please find the improved text in between lines 113-115. Thank you so much.

Line 113-115: Prior to data collection, selected communities were visited and with the permission of the community leaders, meetings were held with the members of the community to intimate them about the purpose of the study and the procedures to be adopted.

C10: The sub title “Collection of faecal samples and parasitological diagnosis” be replaced with “ Faecal samples collections and processing”.

R10: We have modified our text as suggested. Please find the improved text in line 130. Thank you

Line 130: **Faecal samples collections and processing**

C11: Line 3-4: The sentence “All participants were asked to provide a sufficiently large stool sample (at least 5 g)” should be replaced with “Individuals who consented to take part in the study were requested to provide stool up to half (about 5gm) into the labelled plastic bottles provided.

R11: We have modified our text as suggested. Please find the improved text in between lines 133-135. Thank you so much.

Lines 133-135: Individuals who consented to take part in the study were requested to provide stool up to half (about 5gm) into the labelled plastic bottles provided.

C12: Line 6: Delete the word “Precisely”.
R12: Deleted as suggested. Please check Line 137

C13: Line 7-8: The sentence “The bottle was covered and agitated vigorously to suspend the stool efficiently in the solution” be replaced with “The bottle was covered and shaken vigorously to form a cloudy suspension”.

R13: We have modified our text as suggested. Please find the improved text in between lines 138-139. Thank you so much.

Line 138-139: The bottle was covered and shaken vigorously to form a cloudy suspension. The stool suspension……

C14: Line 9: Delete the word “further”

R14: Deleted as suggested.

Line 139: The stool suspension was strained into…..

C15: Line 11: The sentence “The supernatant was also discarded after centrifuging” be replaced with “The supernatant was decanted”.

R15: We have modified our text as suggested. Please find the improved text in line 141. Thank you

Line 141: The supernatant was decanted

C16: Line 12-14: The sentence “7 ml of normal saline was later added to the sediment, after which 3 ml of ether was finally added to the suspension”. ( I did not agree with this procedure, it is faulty). The deposit or residue obtained after decanting the supernatant should be resuspended in distilled water or saline of a known vol and shaking vigorously. Thereafter, an equal vol of ether will then be added (i.e if 3ml of distilled water or saline was used to resuspend the residue, 3ml of ether should then be added). The authors should correct this procedure.

R16: Thank you for this explanation. However, we followed the procedure describe by Endriss *et al.* 2005. We have included the reference immediately after the text. Line 143

**Results**

Sex and age distribution of study participants

C17: The sentence “A total of 1,499 respondents were subsequently enrolled after consent across the 33 communities and 20 LGAs. However, only 1,027 (68.5%) of them consented to the provision of adequate stool samples (~5g) for laboratory analysis for parasites’ ova or larva. By demography, 899(60.0%) were females and 600(40.0%) were males” should be rewritten as “ A total of 1499 participants comprising of 899 (60%) females and 600 (40%) males were enrolled across the 33 communities in 20LGAs out of which 1024 (68.5%) returned stool samples for laboratory analysis.

R17: We have modified our text as suggested. Please find the improved text in between lines 166-168. Thank you so much.

Line 166-168: A total of 1499 participants comprising of 899 (60%) females and 600 (40%) males were enrolled across the 33 communities in 20LGAs out of which 1024 (68.5%) returned stool samples for laboratory analysis.

C18: The sentence “The sex ratio of females to males was 3:2. In addition, the highest number of respondents were within the age range 41-70yrs (40.6%), followed by 26-40yrs (30.3%), 5-15yrs (10.7%), 16-25yrs (10.4%) and >70yrs (7.0%) (Table 2)” should be re-written as “The ages of the enrolled participants are provided in table 2.

R18: We have modified our text as suggested. Please find the improved text in between lines 168-169. Thank you so much.

Line 168-169: The ages of the enrolled participants are provided in table 1.


**C19-24: Co-distribution patterns of Ascaris, Trichuris and Hookworm infections in Ogun State**

C19: Line1: Replace the words “mono infection” with single infection

C20: Line 2: Delete the word “only”

C21: Line 3: Replace the word “co-distribution” with the word “combination”

C22: Line 5: Begin with combination of Ascaris and Trichuris and delete the word “co-distribution”

C23: Line 6 & 7: Rewrite “while Trichuris and Hookworm co-distribution was recorded in 6 LGAs;” as “while combination of Trichuris and hookworm was……………………….”

C24: Line 8-10: Rewrite “However, for triple infections, the three species (Ascaris, Trichuris and Hookworm) were co-distributed in 6 LGAs; Ado-Odo, Ipokia, Obafemi-Owode, Ogun waterside, Yewa North and Yewa South” as “ Triple infection comprising Ascaris, Trichuris and hookworm was recorded in 6 LGAs (table 3)”.

R19-24: We have considered all the corrections (C19-24) and modified our text accordingly to reflect them. Please find the improved text in between lines 178-187. Thank you so much

**Line 178-187:**

**Co-distribution patterns of *Ascaris*, *Trichuris* and Hookworm infections in Ogun State**

Figure 3 shows the co-distribution patterns of STH infections in the State. Single infection was recorded for *Ascaris lumbricoides* in 3 LGAs; Ewekoro, Ikenne and Ijebu northeast. For double infections, combination of *Ascaris* and Hookworm were the most predominant, observed in 15 LGAs except Ewekoro, Ijebu east, Ijebu northeast, Ikenne and Ijebu ode. Combination of *Ascaris and Trichuris* co-distribution was recorded in 7 LGAs; Yewa north, Yewa south, Ado-odo ota, Ijebu east, Ipokia, Obafemi owode and Ogun waterside, while combination of *Trichuris* and hookworm was recorded in 6 LGAs; Ado-odo ota, Ipokia, Obafemi owode, Ogun waterside, Yewa north and Yewa south. Triple infections comprising *Ascaris*, *Trichuris* and hookworm was recorded in 6 LGAs (Table 3).

Aggregated prevalence of Ascaris, Trichuris and Hookworm infections in Ogun State
C25: The sentences “The aggregated prevalence of 17.2% was recorded for at least one infection of Ascaris lumbricoides, Trichuris trichiura or hookworm. The prevalence range was between 5.3 – 49.2% across the LGAs (Figure 4). Infections were highest in Yewa south, lowest in Yewa north and no infection was recorded in Ijebu-Ode. Of the 19 endemic LGAs, 9 had prevalence
status ranging between 20.0%-49.2% (Abeokuta north, Abeokuta south, Ifo, Ikenne, Ipokia, Obafemi owode, Ogun waterside, Remo north and Yewa south” should be reduced to “ The highest prevalence of 49.2% was recorded in Yewa South while the lowest of 5.3% was recorded in Yewa North, however, no infection was recorded in Ijebu-Ode.

R25: We have considered the corrections and modified our text accordingly to reflect them. Please find the improved text in between lines 194-195.

The highest prevalence was recorded in Yewa South while the lowest was recorded in Yewa North, however, no infection was recorded in Ijebu-Ode (Figure 4).

C26: Delete this sentence “However, 10 LGAs haprevalence status ranging between 5.3%-15.8% which is below the WHO recommended thresholds for preventive chemotherapy (Ado-odo ota, Ewekoro, Ijebu east, Ijebu north, Ijebu north east, Imeko afon, Odeda, Odogbolu, Sagamu and Yewa north).

R26: We have considered the comments raised. We believe deleting this sentence will mask the pattern we are trying to showcase. Therefore we have modified our text accordingly to reflect them. Please find the improved text in between lines 196-199.

Of the 19 endemic LGAs, 9 had prevalence status ranging between 20.0%-49.2% and 10 LGAs had prevalence status ranging between 5.3%-15.8%. There were significant differences in the prevalence record for any STH species across the 19 endemic LGAs (P=0.000) (Figure 4).

Specific prevalence estimates for Ascaris, Trichuris and Hookworm infections in Ogun State.

C27: The contents under this section is too wordy. The authors should highlight significant findings in terms of the most frequently encountered parasite in each LGA, the range of other parasites and if there is significant differences in prevalence of infection across the communities.

R27: We have considered the suggestions raised and modified our text accordingly to reflect them. Please find the improved text in between lines 201-211

**Specific prevalence estimates for *Ascaris*, *Trichuris* and Hookworm infections in Ogun State.**

By species’ prevalence*,* an overall prevalence of 13.6% was recorded for *Ascaris lumbricoides,* followed by hookworm with 4.6% and *Trichuris trichiura* with 1.7% (Table 3). The lowest prevalence for the three infections were observed in Yewa north., while Abeokuta south, Obafemi owode and Ipokia recorded the highest prevalence for ascariasis*,* hookworm infections and trichuriasis respectively (Table 3). Spatial-wise, *Ascaris lumbricoides was* the most predominant, found in 28(84.8%) communities (Figure 6), followed by hookworm in 19 (57.6%) communities (Figure 7) and *Trichuris trichiura* in 9(27.3%) communities. There were significant differences in the prevalence record for each parasitic infection across the endemic LGAs (P=0.000).

.

Aggregated and specific mean intensity estimates for Ascaris, Trichuris and Hookworm infections in Ogun State.

C28: The authors should express the intensity as eggs per gram (epg) of faeces i.e epg should be put in front of the mean intensity values in all cases.

R28: We have considered the suggestions raised and modified our text accordingly to reflect them. Please find the improved text in between lines 226-239

Line 226-239

**Aggregated and specific mean intensity estimates for *Ascaris*, *Trichuris* and Hookworm infections in Ogun State.**

Table 4 shows the intensity estimates for *Ascaris*, *Trichuris* and hookworm infections in Ogun state. The aggregated geometric mean intensity of infections was 0.14$\pm$0.01epg with mean intensity ranging from 0.03$\pm$0.01epg to 0.43$\pm$0.06epg across the LGAs. The aggregated intensity shows that worm loads were highest in Obafemi owode and lowest in Imeko-afon. By species intensities*, Ascaris lumbricoides* infection intensity was the highest with, 0.11$\pm$0.01epg, followed by hookworm with 0.03$\pm$0.01epg and *Trichuris trichiura* with 0.01$\pm$0.00epg. *Ascaris* mean intensities range between 0.01$\pm$0.01epg and 0.32$\pm$0.05epg, with the lowest in Imeko afon and highest in Ifo and Yewa south. For Hookworm infection, intensities range between 0.01$\pm$0.00epg and 0.13$\pm$0.04epg, with the lowest in Ijebu north, Yewa north, Yewa south, Ogun waterside, Imeko afon and highest in Obafemi-owode. *Trichuris* mean intensities range between 0.01$\pm$0.01epg and 0.10$\pm$0.05epg with the highest load in Ipokia and the lowest in Ogun waterside, Ijebu east and Yewa north.

C31: The authors stated under this section about the single, double and triple infections, however there was no dat presented to show the prevalences of this infection. The table should be provided.

R31: A venn diagram was used to present the number of LGAs where single, double and triple infections could be found. Please see figure 3. We didn’t report specific combination prevalences in our text to reduce the number of tables.

**Discussion.**

C32: The authors should use simple grammar to discuss their findings.

R32: We have re-worked our discussion to enhance clarity. Please check Line 277-290

6. PLOS authors have the option to publish the peer review history of their article ([what does this mean?](https://journals.plos.org/plosone/s/editorial-and-peer-review-process#loc-peer-review-history)). If published, this will include your full peer review and any attached files.


If you choose “no”, your identity will remain anonymous but your review may still be made public.


**Do you want your identity to be public for this peer review?** For information about this choice, including consent withdrawal, please see our [Privacy Policy](https://www.plos.org/privacy-policy).

Reviewer #1: No

Reviewer #2: No


[NOTE: If reviewer comments were submitted as an attachment file, they will be attached to this email and accessible via the submission site. Please log into your account, locate the manuscript record, and check for the action link "View Attachments". If this link does not appear, there are no attachment files to be viewed.]


While revising your submission, please upload your figure files to the Preflight Analysis and Conversion Engine (PACE) digital diagnostic tool, [https://pacev2.apexcovantage.com/](https://pacev2.apexcovantage.com/). PACE helps ensure that figures meet PLOS requirements. To use PACE, you must first register as a user. Registration is free. Then, login and navigate to the UPLOAD tab, where you will find detailed instructions on how to use the tool. If you encounter any issues or have any questions when using PACE, please email us at [figures@plos.org](mailto:figures@plos.org). Please note that Supporting Information files do not need this step.
