# Supplementary material for: Distribution of ascariasis, trichuriasis and hookworm infections in Ogun State, Southwestern Nigeria
Source: PLoS One. 2020 Jun 8;15(6):e0233423. doi: 10.1371/journal.pone.0233423 (PMC7279584; doi:10.1371/journal.pone.0233423)
Supplement: Attachment — Submitted filename: Response to Reviewers comments.docx [file pone.0233423.s004.docx]

**Reviewer #2: Comments on the revised version of the MS**

General comments: The authors revised the MS in line with the suggestions provided, however, there are still some minor corrections that needs to be effected in the discussion section.

**Specific comments**

Discussion

**C1:** Line 283-284: The prevalence estimates also corroborates ………………………………….due to chemotherapy programmes in SSA countries (The statement is not clear). However, I would suggest that the sentence should be re-written to read “The prevalence values for the 3STHs recorded in this study is comparable ………………………………. (The values obtained should be compared with the findings of previous studies and may then give reason for the similarities or differences.).

**R1:** The corrections have been made as suggested. We have compared our prevalence values with three reports (2 from Nigeria and one from Ethiopia (Gemechu et al. 2020)

Please find our improved text between Line 283-287

The prevalence values of the three soil transmitted helminth (STH) infections recorded in this study is comparable with the findings of Mogaji *et al.* [2], Oluwole *et al.* [12] and Gemechu et al. [14]. The similarities observed supports existing presumptions on decreasing trend of STH infections due to on-going chemotherapy programmes in most endemic SSA countries [15,16].

**C2:** Line 287: Replace the words “Sub-state level” with LGAs and communities

R2: This correction has been made (Line 289). Thank you

**C3:** Line 292: Replace the following words “reported” with “recorded”; “corroborates” with “comparable”; “recent” with “previous”.

**R3:** This correction has been made.

Line 293-294: The prevalence recorded for *Ascaris lumbricoides* infections in this study is comparable with findings from previous epidemiological surveys [12,17].

**C4:** Line 293-295: The sentence “Ascaris lumbricoides infections were observed in …………………………………..by Oluwole et al. (12) should be recast to read “The increase in prevalence of A. lumbricoides infection as observed in 19 of the 20 LGAs studied is similar to the findings of Oluwole et al (12) with respect to spatial pattern of A. lumbricoides. This may be due to similar ecological factors (mention the factors) which favours the transmission of STHs in the areas of study”.

**R4:** The corrections have been made as suggested (Line 294-298)

The increase in prevalence of *A. lumbricoides infection* as observed in 19 of the 20 LGAs studied is similar to the findings of Oluwole et al [12] with respect to spatial pattern of *A. lumbricoides*. This may be due to similar ecological factors such as soil moisture, pH or temperature, which favours the transmission of STHs in the areas of study.

**C5:** Line 296-299: Please delete the sentences “where moderate……………………………………… of STH parasites in the state”.
**R5:** The sentences have been deleted. Thank you

**C6:** Line 300-302: The sentences “However, none of the LGAs studied has……………………. Population in the report of Oluwole et al. (12)” should be recast to read “The moderate prevalence of less than 20% recorded in various communities in contrast with prevalence of more than 50% reported by Oluwole et al. (12) may be due to the difference in the composition of population studied.
**R6:** The corrections have been made as suggested (Line 298-300)

The moderate prevalence of less than 20% recorded in various communities in contrast with prevalence of more than 50% reported by Oluwole et al. [12] may be due to the difference in the composition of population studied.

**C7:** Line 313: Please delete the word “aggregate”
**R7:** The word has been deleted. Thank you

**C8:** Line 316: Your assertion that hookworms egg can withstand extreme temp (I guess you meant extreme heat). Remember hookworm eggs have thin shells compared with thick shell of Ascaris eggs. It is only Ascaris eggs which can withstand adverse environmental conditions.
**R8: Thank you for this comment. We have removed the line entirely to improve on clarity**

**C9:** Line 317: Please replace the word ‘commonalities” with the word “similarities”
**R9:** The corrections have been made as suggested (Line 313)

**C10:** Line 326: replace the word “observed” with “reported”
**R10:** The corrections have been made as suggested (Line 317)

**C11:** Line 332: Replace the word “study” with “studied”
**R11:** The corrections have been made as suggested

**C12:** Table 3: The title should be rephrased to read “Intensity (Mean ± SEM) of Ascaris, hookworm and Trichuris infection in selected population in Ogun State”.
**R12:** The corrections have been made as suggested (Line 253)

**C13:** In table 3: Re-write the abbreviation “SE” as “SEM”. Define SEM as Standard Error of Mean
**R13:** The corrections have been made as suggested (Line 253-256)

**C14:** Table 4: The title should be rephrased to read "Prevalence of STH infections in relation to sex and age in selected population

**R14:** The corrections have been made as suggested (Line 264-266)

**C15:** Still in Table 4: Under Age group insert the word "Years" and delete the word "Years" from each age group.

**R15:** The corrections have been made as suggested (Line 264-266)
